# Gentisyl Alcohol Inhibits Proliferation and Induces Apoptosis via Mitochondrial Dysfunction and Regulation of MAPK and PI3K/AKT Pathways in Epithelial Ovarian Cancer Cells

**DOI:** 10.3390/md17060331

**Published:** 2019-06-03

**Authors:** Jiyeon Ham, Whasun Lim, Kyeongwon Kim, Young Mok Heo, Seung Mok Ryu, Dongho Lee, Jae-Jin Kim, Gwonhwa Song

**Affiliations:** 1 Department of Biotechnology, College of Life Sciences and Biotechnology, Korea University, Seoul 02841, Korea; glorijy76@korea.ac.kr; 2 Department of Food and Nutrition, College of Science and Technology, Kookmin University, Seoul 02707, Korea; wlim@kookmin.ac.kr; 3 Division of Environmental Science & Ecological Engineering, College of Life Sciences & Biotechnology, Korea University, Seoul 02841, Korea; rudndjs@korea.ac.kr (K.K.); hym011@korea.ac.kr (Y.M.H.); 4 Herbal Medicine Resources Research Center, Korea Institute of Oriental Medicine, Naju-si, Jeollanam-do 58245, Korea; smryu@kiom.re.kr; 5 Department of Biosystem and Biotechnology, College of Life Sciences & Biotechnology, Korea University, Seoul 02841, Korea

**Keywords:** ovarian cancer, gentisyl alcohol, mitochondrial dysfunction, calcium dysregulation

## Abstract

Ovarian cancer is one of the prevalent gynecological cancers occurring in women. In particular, the efficiency of standard therapeutic methods decreases when recurrence and chemoresistance ensue. To assist standard anti-cancer agents in the cure of ovarian cancer, development and application of new compounds such as small molecules or natural products are required. Gentisyl alcohol is one of the secondary metabolites that can be obtained by purification from bacteria or fungi and is known to have antibacterial, antifungal, antiviral, and anti-cancer effects. In the present study, we verified the effect of gentisyl alcohol derived from marine *Arthrinium* sp. on suppressing proliferation and inducing apoptosis via DNA fragmentation in human ovarian cancers cells (ES2 and OV90 cells). We also confirmed that there was an accumulation of sub-G1 cells and a loss of mitochondrial membrane potential with calcium dysregulation in gentisyl alcohol-treated ovarian cancer cells. Moreover, gentisyl alcohol up-regulated signal transduction of MAPK and PI3K/AKT pathways. Collectively, our results demonstrated the possibility of gentisyl alcohol as a novel therapeutic agent for human ovarian cancer.

## 1. Introduction

Ovarian cancer is one of the most fatal and common gynecological tumors, which is the fifth leading cause of cancer-related mortality in women [1]. In this study, we dealt with human epithelial ovarian ES2 (clear cell carcinoma) and OV90 (high-grade serous carcinoma) cells, the cancers of which occupy about 10% and 70% of incidence in epithelial ovarian cancers, respectively [2]. Due to these limitations of early diagnosis, most of the patients reach an advanced stage of cancer, and the five-year survival rate drops to less than 30% [3]. Furthermore, ovarian cancer shows a high rate of recurrence (over 50%) and resistance to platinum-based chemotherapy (about 25%), especially in epithelial ovarian cancers [4,5]. Therefore, efforts are being made to investigate novel therapies, such as targeting angiogenesis, or treating with poly (ADP-ribose) polymerase (PARP) inhibitors or with bevacizumab, for chemotherapy-resistant and recurrent ovarian cancers [5].

Gentisyl alcohol is one of the phenolic alcohols extracted from fungi and has biological and pharmaceutical properties, such as antibacterial effects [6]. In addition, it is reported to have anti-cancer activity in several cancers, as well as antiviral activities and antioxidant properties [7]. However, the effects of gentisyl alcohol on human ovarian cancer have not yet been elucidated. In this study, our hypothesis is that gentisyl alcohol may enhance apoptosis against progression of ovarian cancer cells. To support our hypothesis, we first demonstrated the effects of gentisyl alcohol on ovarian cancer cells (ES2 and OV90 cells) as follows: (1) proliferative activity; (2) cell death with cell-cycle arrest; (3) unsettlement of mitochondrial membrane potential and hampering calcium homeostasis; and (4) intracellular signal transduction with or without pharmacological inhibitors.

## 2. Results

### 2.1. Gentisyl Alcohol Inhibits Proliferation of Ovarian Cancer ES2 and OV90 Cells

**Figure 1:**
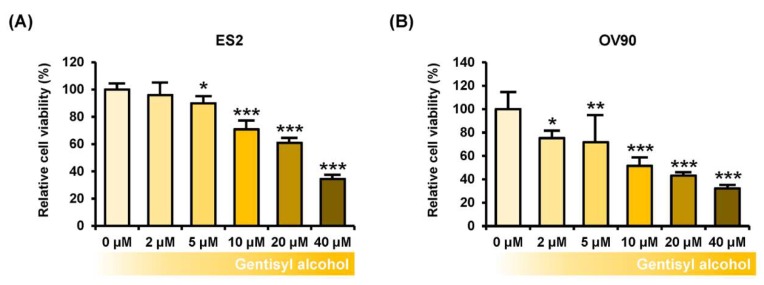

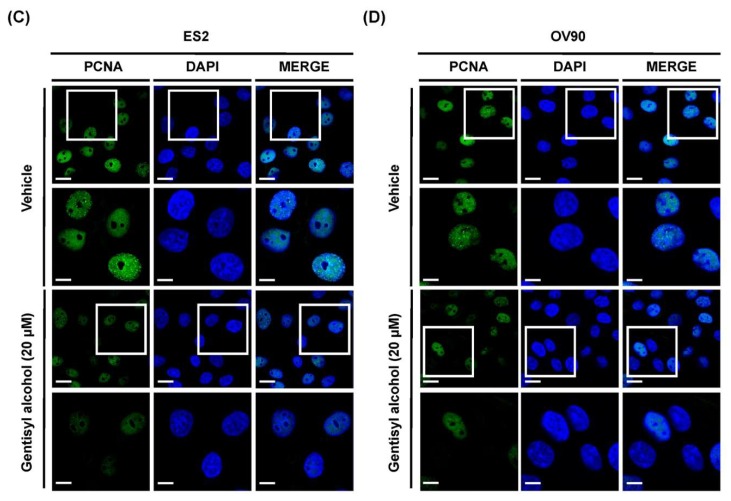
Effects of gentisyl alcohol on suppressing proliferation in human ovarian cancer cells. (**A**,**B**) Proliferation assays were performed on gentisyl alcohol-treated ES2 and OV90 cells in dose-dependent manners. The results are presented as a bar graph indicating percentages relative to vehicle-treated cells as the control. (**C**,**D**) Microscopy photo of ES2 and OV90 cells expressing proliferating cell nuclear antigens (PCNAs) emitting green fluorescence in the nuclei. 4′,6-diamidino-2-phenylindole (DAPI, blue) was used to counter-stain the nuclei co-localized with PCNAs. The scale-bar represents 20 μm (the first and third horizontal panels) and 10 μm (the second and fourth horizontal panels). Asterisks indicate significant effects on treated cells compared to those on vehicle-treated cells (* *p* < 0.05, ** *p* < 0.01, and *** *p* < 0.001).

To measure the effects of gentisyl alcohol on suppressing proliferation of human ovarian cancer cells, we performed proliferation assays on ES2 and OV90 cells treated with gentisyl alcohol (0 μM, 2 μM, 5 μM, 10 μM, 20 μM, and 40 μM) in a dose-dependent manner. Our results revealed that gentisyl alcohol suppressed the proliferative activities of ES2 by up to 39% and OV90 by up to 57% at the concentration of 20 μM, as compared to vehicle-treated cells (100%) (Figure 1A,B). In addition, we also detected expression of proliferating cell nuclear antigens (PCNA), which is commonly used as a proliferation marker and emits green fluorescence in immunostained ES2 and OV90 cells (Figure 1C,D). After treatment with gentisyl alcohol, the intensity of green fluorescence was reduced in both the ES2 and OV90 cells. Based on these results, we confirmed that gentisyl alcohol suppresses the proliferation of human epithelial ovarian cancer cells.

### 2.2. Gentisyl Alcohol Induces Apoptosis and an Accumulation of Sub-G1 in Ovarian Cancer Cells

**Figure 2:**
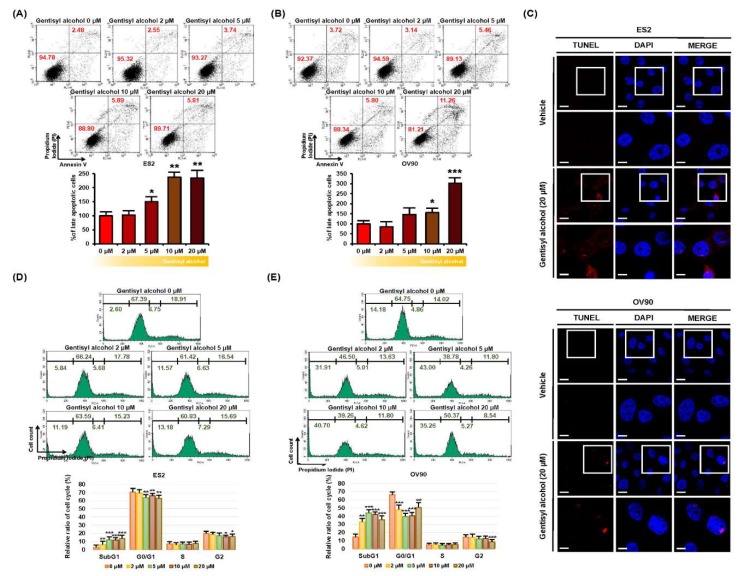
Effects of gentisyl alcohol on inducing apoptosis in human ovarian cancer ES2 and OV90 cells. (**A**,**B**) Detection of gentisyl alcohol-induced apoptosis on ES2 and OV90 cells were measured by flow cytometry using annexin V and propidium iodide (PI). The population of ovarian cancer cells located in the upper right panel of the quadrant is of late apoptotic cells and they are analyzed as a percentage relative to vehicle-treated cells. (**C**) Apoptotic DNA fragments were labeled with red fluorescence in the terminal deoxynucleotidyl transferase dUTP nick end labeling (TUNEL) immunocytochemical studies. Nuclei were counter-stained with DAPI (blue) for co-localization. The scale-bar indicates 20 μm (the first/third horizontal panels) and 10 μm (the second/fourth horizontal panels). (**D**,**E**) Each phase of the cell cycle was analyzed by staining with PI using flow cytometry on gentisyl alcohol-treated ES2 and OV90 cells. Asterisks represent significant effects of treatment (* *p* < 0.05, ** *p* < 0.01, and *** *p* < 0.001).

Next, we investigated whether gentisyl alcohol triggers cell death in ovarian cancer cells using annexin V and propidium iodide (PI) staining. As illustrated in Figure 2A,B, the population of ES2 and OV90 cells in the upper right quadrant (indicating late apoptosis) increased by 234% and 303%, respectively, in response to gentisyl alcohol (20 μM) compared to vehicle-treated cells. We also performed the terminal deoxynucleotidyl transferase dUTP nick end labeling (TUNEL) assay (one of the hallmarks of apoptosis) to detect DNA fragmentation on gentisyl alcohol (20 μM)-treated ES2 and OV90 cells using the terminal deoxynucleotidyl transferase (TdT) enzyme reaction (Figure 2C). A higher intensity of red fluorescence was detected in the nuclei of gentisyl alcohol-treated ES2 and OV90 cells than in vehicle-treated cells. Also, gentisyl alcohol induced an accumulation of sub-G1 cells in ES2 and OV90 cells, whereas the populations of G0/G1 and G2 phase cells decreased (Figure 2D,E). These results revealed that gentisyl alcohol induced apoptosis and showed an increase in cell population at the sub-G1 in human epithelial ovarian cancer ES2 and OV90 cells.

### 2.3. Gentisyl Alcohol Disrupts Mitochondrial Function and Calcium Homeostasis of ES2 and OV90 Cells

**Figure 3:**
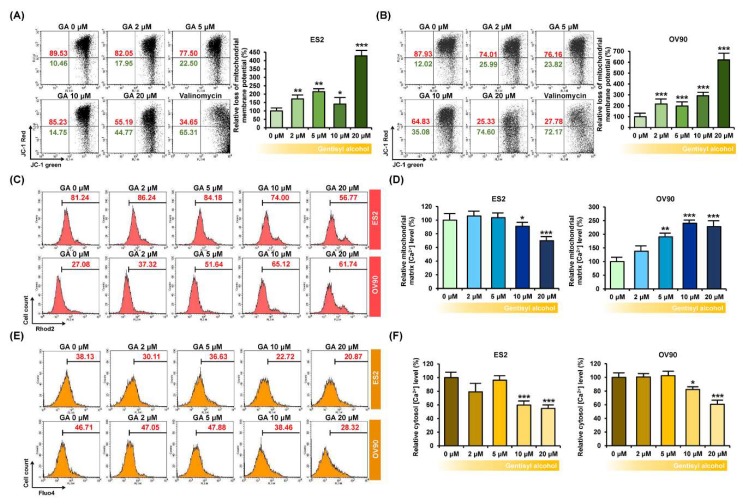
Effects of gentisyl alcohol on unsettlement of mitochondrial membrane potential (MMP) and disruption of calcium homeostasis in ovarian cancer cells. (**A**,**B**) Flow cytometry detected MMP loss of ovarian cancer cells in response to gentisyl alcohol using 5,5′,6,6′-tetrachloro-1,1′,3,3′-tetraethylbenzimidazolcarbocyanine iodide (JC-1) dye. The population of the upper right panel to the lower right panel of the quadrants is presented as a percentage relative to vehicle-treated ovarian cancer cells. (**C**,**D**) Rhod2 fluorescence of ovarian cancer cells in response to gentisyl alcohol was detected by flow cytometry and is presented as a percentage relative to the vehicle. (**E**,**F**) Relative cytosolic calcium levels were detected using Fluo4 dye and measured by flow cytometry. Bar graphs are presented as a percentage relative to the vehicle. Asterisks indicate significant effects on treated cells compared to those on vehicle-treated cells (* *p* < 0.05, ** *p* < 0.01, and *** *p* < 0.001).

To confirm the functional effects of gentisyl alcohol on ovarian cancer cells, we measured the mitochondrial membrane potential (MMP, ΔΨ) using JC-1 dye, which is converted from a green monomer to a red aggregate in line with the MMP function. Our results indicated that the JC-1 monomer in gentisyl alcohol-treated ES2 and OV90 cells was increased by approximately 428.0% and 620.6%, respectively, at 20 μM of gentisyl alcohol (Figure 3A,B). Moreover, when we measured the relative mitochondrial matrix calcium ion concentration ([Ca^2+^]_m_) using Rhod2, which is selectively accumulated in mitochondria, it gradually decreased in response to gentisyl alcohol in ES2 cells but increased in OV90 cells (Figure 3C,D). In contrast, the relative cytosolic calcium concentration ([Ca^2+^]_i_), detected by Fluo4, was diminished by gentisyl alcohol in both the ES2 as well as OV90 cells (Figure 3E,F). Therefore, these data suggested that gentisyl alcohol perturbed the mitochondrial function of human ovarian cancer cells, leading to disruption of MMP and calcium homeostasis.

### 2.4. Gentisyl Alcohol Activates ERK1/2 and P38 Phosphorylation and Inactivates AKT Phosphorylation in ES2 and OV90 Cells

**Figure 4:**
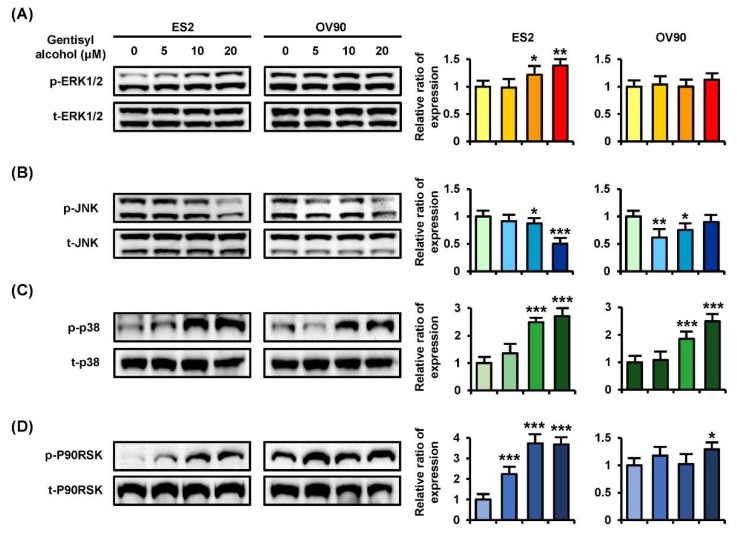
Phosphorylation changes of the MAPK pathway induced by gentisyl alcohol in dose-dependent manners on human ovarian cancer cells. Phosphorylation of (**A**) ERK1/2, (**B**) JNK, (**C**) P38, and (**D**) P90RSK in gentisyl-treated ES2 and OV 90 cells was analyzed by Western blotting. Each immunoblot was analyzed relative to the total proteins for normalization of values. Asterisks indicate significant effects on treated cells compared to naïve cells (* *p* < 0.05, ** *p* < 0.01, and *** *p* < 0.001).

**Figure 5:**
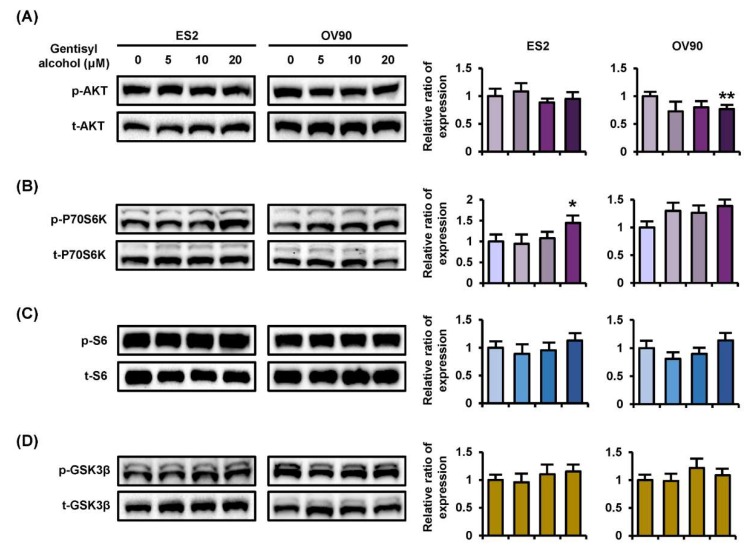
Phosphorylation changes of the PI3K pathway induced by gentisyl alcohol in dose-dependent manners on human ovarian cancer cells. Phosphorylation of (**A**) AKT, (**B**) P70S6K, (**C**) S6, and (**D**) GSK3β in gentisyl-treated ES2 and OV 90 cells was analyzed by Western blotting. Each immunoblot was analyzed relative to the total proteins for normalization of values. Asterisks indicate significant effects on treated cells compared to naïve cells (* *p* < 0.05 and ** *p* < 0.01).

To confirm the cellular mechanisms of MAPK and PI3K/AKT signaling pathways, we performed Western blot analysis on ES2 and OV90 cells in response to gentisyl alcohol (0 μM, 5 μM, 10 μM, and 20 μM). Phosphorylation of ERK1/2 (p-ERK1/2) and P38 MAPK were increased in both ES2 and OV90 cells in dose-dependent manners (Figure 4A,C). However, the p-JNK was reduced in both the gentisyl alcohol-treated ovarian cancer cells (Figure 4B). As a downstream signaling molecule of ERK1/2, the p-P90RSK was increased by gentisyl alcohol in a dose-dependent manner (Figure 4D). In the case of the PI3K/AKT pathway, we determined the activation of p-P70S6K, wherein the p-AKT was slightly decreased by gentisyl alcohol in both ovarian cancer cell types (Figure 5A,B). In addition, the p-S6 and p-GSK3β did not change significantly with the gentisyl alcohol treatment in both the cell types (Figure 5C,D). These results suggested that the changes caused by gentisyl alcohol-induced PI3K/AKT pathway were not canonical. Collectively, gentisyl alcohol induced the activation of ERK1/2 and P38 MAPK and non-canonical PI3K/AKT pathways in human ovarian cancer cells.

### 2.5. Effects of Gentisyl Alcohol on Changes of MAPK and PI3K/AKT Signaling Molecules with or without Pharmacological Inhibitors

**Figure 6:**
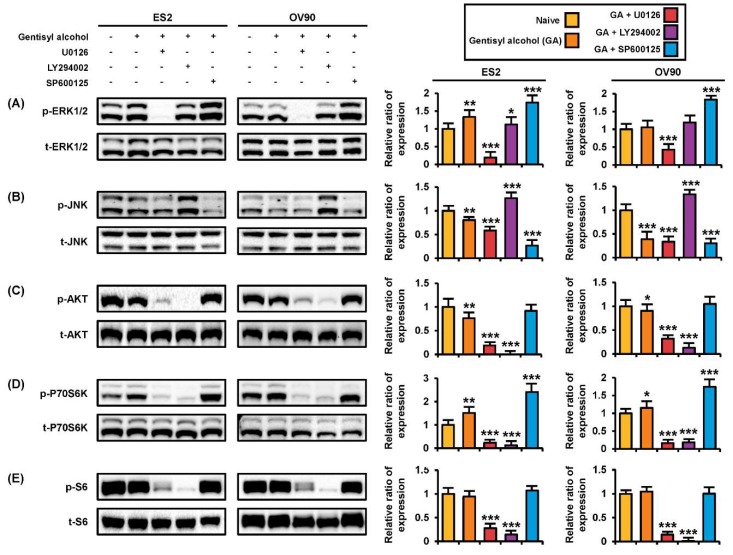
Changes in protein kinase activities on human ovarian cancer cells by co-treatment of gentisyl alcohol and pharmacological inhibitors. (**A**–**E**) ES2 and OV90 cells were pre-treated with U0126 (20 μM), LY294002 (20 μM), and SP600125 (20 μM) for 2 h prior to 20 μM of gentisyl alcohol treatment. Immunoblots were analyzed relative to the total proteins for normalization of values. Asterisks indicate significant effects on treated cells compared to naïve cells (* *p* < 0.05, ** *p* < 0.01, and *** *p* < 0.001).

To verify the mode of action of gentisyl alcohol, we conducted Western blot analysis with proteins extracted from ES2 and OV90 cells treated with U0126 (an ERK1/2 inhibitor), LY294002 (a PI3K/AKT inhibitor), or SP600125 (a JNK inhibitor) prior to incubation with gentisyl alcohol (Figure 5). Gentisyl alcohol-induced phosphorylation of ERK1/2 was restored to basal levels by LY294002, whereas SP600125 upregulated the p-ERK1/2 levels in ES2 and OV90 cells (Figure 6A). However, the p-ERK1/2 was completely blocked by U0126 in both cell types. The p-JNK was further suppressed by U0126 and SP600125 relative to treatment with gentisyl alcohol only but was elevated by LY294002 in both the ES2 and OV90 cells (Figure 6B). In the PI3K/AKT pathways, treatment with a combination of gentisyl alcohol with either U0126 or LY294002 almost completely blocked the phosphorylation of AKT, P70S6K, and S6 proteins in both ovarian cell types (Figure 6C–E). In the case of p-P70S6K, the combined treatment of SP600125 with gentisyl alcohol resulted in higher phosphorylation of P70S6K than with gentisyl alcohol alone in both ES2 and OV90 cells (Figure 6D). Based on these results, we can deduce that gentisyl alcohol changes signal transduction in the MAPK and PI3K/AKT pathways of human ovarian cancer cells.

## 3. Discussion

**Figure 7:**
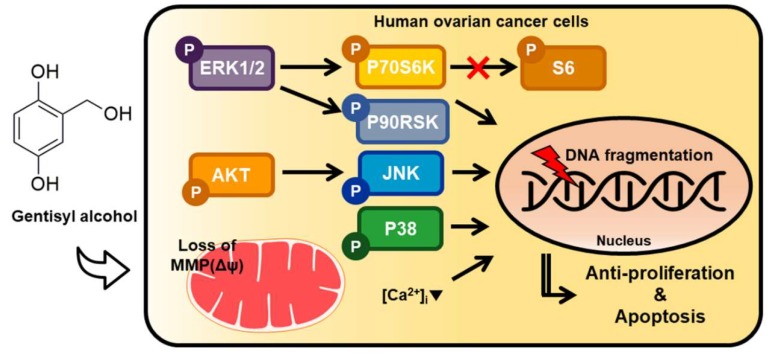
Illustration of hypothetical mechanisms induced by gentisyl alcohol on human ovarian cancer ES2 and OV90 cells. Regulation of MAPK and PI3K/AKT pathways are indicated by arrows. (Δψ: mitochondrial membrane potential; [Ca^2+^]_i_: intracellular calcium ion concentration).

In the present study, we confirmed the effects of gentisyl alcohol on ovarian cancer cells (ES2 and OV90 cells) due to its anti-proliferative and apoptotic activities, which perturbed mitochondrial function and calcium homeostasis by changing MAPK and PI3K/AKT signaling, as illustrated in Figure 7. Based on our results, we suggest that gentisyl alcohol may be a novel anti-cancer supplement or agent for treating ovarian cancers.

Marine-derived fungi, such as *Arthrinium* spp., *Aspergillus* spp., *Cephalosporium* spp., and *Fusarium* spp., are crucial sources of secondary bioactive metabolites for applications in drug discovery [8,9]. Particularly, functional compounds possessed by the *Arthrinium* spp. prevent the formation of bacterial biofilms and oxidative stress by regulating reactive oxygen species [10]. However, the activities of *Arthrinium* spp. have not been widely elucidated until now. The gentisyl alcohol used in this research was isolated from secondary metabolites of marine *Arthrinium* spp. [10]. Although it has been found in *Penicillium terrestre* and *Phoma herbarum* endophytic in *Curcuma longa* L., gentisyl alcohol is not a major compound of them [11,12]. There are also ways to biosynthesize gentisyl alcohol via engineered *Escherichia coli*. [13]. Gentisyl alcohol has already been reported to inhibit cell proliferation in human breast and colon cancer and its derivatives have anti-cancer effects on lung carcinoma and leukemia cell lines [11]. One of the derivatives of gentisyl alcohol inhibits some of the tyrosine kinase family members, such as tyrosine kinase inhibitors (TKIs) [11]. In the case of ovarian cancers, TKI treatments could show good therapeutic efficiency via the inhibition of protein tyrosine kinases [14]. As demonstrated by our studies, we determined the anti-proliferative and apoptotic effects of gentisyl alcohol derived from *Arthrinium* spp. on human ovarian cancer cells. Thus, our study can be considered novel research in the development of marine-derived bioactive metabolites as therapeutics for ovarian cancer.

Mitochondria function and calcium homeostasis are crucial for regulating intracellular signal transduction and metabolism [15]. The upregulation of mitochondrial and cytosolic calcium through treatment with the pharmacological inhibitor ABT737 induces mitochondria-mediated apoptosis by inducing endoplasmic reticulum (ER) stress in cisplatin-resistant ovarian cancer [16]. However, when calcium signaling was blocked, generation of oxidative stress did not occur and ovarian cancer cells demonstrated resistance to initial chemotherapy [17], implying that calcium homeostasis and mitochondria function are important for ovarian cancer. For example, in chemotherapy-resistant ovarian cancer, cells survive by inhibiting the imbalance of mitochondrial calcium homeostasis and by inhibiting ER stress [18]. Generally, mitochondria-mediated apoptosis is caused by mitochondrial calcium overload and the formation of a complex between Bax and Bak proteins to release cytochrome *c* [19]. However, our results showed a loss of mitochondrial membrane potential (MMP) with a decline in mitochondrial matrix calcium ion concentration [Ca^2+^]_m_ in ES2 cells and an increase in [Ca^2+^]_m_ in OV90 cells. This might be caused as a result of the malfunction of mitochondrial calcium uniporter (MCU), the function of which is the transport of calcium ions from the ER and cytosol to regulate calcium balance for cellular metabolism [20]. When the MCU is silenced or knocked down, [Ca^2+^]_m_ decreased [21]. This decline of [Ca^2+^]_m_ by MCU silencing results in caspase-independent cell death in breast cancer, regardless of cytosolic calcium concentration [Ca^2+^]_i_ [22]. Also, the inhibition of [Ca^2+^]_m_ leads to ER stress and cytotoxicity in ovarian cancer [23].

Usually, PI3K/AKT pathway, known as a signal of cell-survival, is involved in various cellular metabolic mechanisms, such as apoptosis, proliferation, and cell growth in a number of cancers [24]. Also, the overexpression of the PI3K/AKT pathway and overall survival of ovarian cancer patients reveal an inverse relationship with each other [25]. Moreover, PI3K and ERK pathways were hyper-activated in platinum chemotherapy-resistant ovarian cancer based on a reported microarray of data [26]. Surprisingly, our data showed unexpected results regarding the PI3K/AKT pathways. As shown in Figure 4, we verified the activation of p-P70S6K; however, p-AKT showed decreased activity, which indicates that gentisyl alcohol does not work as a canonical pathway in ES2 and OV90 cells. GSK3β, which usually displays upregulation by inhibition of AKT in ovarian cancer, and the S6 protein, generally known to occur downstream to PI3K/AKT, did not show significant changes with gentisyl alcohol in both ES2 and OV90 cells. The stimulation of Ras/MAPK signaling induces caspase activity, depolarized mitochondrial membranes, and reactive oxygen species (ROS) production, leading to apoptosis in ovarian cancer [27,28]. According to previous results, gentisyl alcohol induced p-P38 protein in both the ES2 and OV90 cells, similar to our results pertaining to the p-ERK1/2 activity. Collectively, the results indicated that gentisyl alcohol changed PI3K/AKT and MAPK pathways during cell death of ovarian cancer cells.

## 4. Material and Methods

### 4.1. Chemicals

Gentisyl alcohol was purified from a culture of *Arthrinium* spp., KUC21332, as described previously [10]. Antibodies against phosphorylated ERK1/2 (Thr^202^/Tyr^204^, catalog number: 9101), JNK (Thr^183^/Tyr^185^, catalog number: 4668), P38 (Thr^180^/Tyr^182^, catalog number: 4511), P90RSK (Thr^573^, catalog number: 9346), AKT (Ser^473^, catalog number: 4060), P70S6K (Thr^421^/Ser^424^, catalog number: 9204), S6 (Ser^235/236^, catalog number: 2211), and GSK3β (Ser^9^, catalog number: 9336). Antibodies against total ERK1/2 (catalog number: 4695), JNK (catalog number: 9252), P38 (catalog number: 9212), P90RSK (catalog number: 9335), AKT (catalog number: 9272), P70S6K (catalog number: 9202), S6 (catalog number: 2217), GSK3β (catalog number: 9315) were purchased from Cell Signaling Technology (Beverly, MA, USA). The inhibitors for ERK1/2 (U0126), PI3K/AKT (LY294002), and JNK (SP600125) were obtained from Enzo Life Sciences, Inc. (Farmingdale, NY, USA).

### 4.2. Cell Culture

ES2 and OV90 human epithelial type of ovarian cancer cells were purchased from the American Type Culture Collection (ATCC; Manassas, VA, USA). For maintaining the cells, we used McCoy’s 5A (modified) medium (catalog number: 16600-082; Invitrogen, Carlsbad, CA, USA) with 10% fetal bovine serum (FBS) and incubated them at 37 °C at an atmosphere of 5% CO_2_. To perform further experiments, ES2 and OV90 cells were incubated in culture medium to 70% confluence in 100 mm tissue culture dishes. Cells were treated with different doses of gentisyl alcohol with or without inhibitors of cell signaling molecules.

### 4.3. Proliferation Assay

ES2 and OV90 cells were seeded into a 96-well plate, and then treated with gentisyl alcohol and various other substances to make the final volume to 100 μL/well. To compensate for the total volume of the vehicle in each well, we added an amount of vehicle up to the highest treated dose for each assay. After 48 h of incubation, we conducted the assay as described previously [29]. 

### 4.4. Immunofluorescence Microscopy

For detecting immunoreactive PCNA expression, ovarian cancer cells were probed with mouse anti-human monoclonal PCNAs (catalog number: sc-56, Santa Cruz Biotechnology, Santa Cruz, CA, USA) at 2 μM. They were then incubated with goat anti-mouse IgG Alexa 488 (catalog number. A-11001, Invitrogen, Carlsbad, CA, USA) at a 1:200 dilution for 1 h at room temperature as described previously [29].

### 4.5. Detection of Apoptotic Cells by Annexin V

Apoptotic cells within the ES2 and OV90 cell lines induced by gentisyl alcohol (0 μM, 2 μM, 5 μM, 10 μM, and 20 μM) after 48 h incubation were analyzed using an fluorescein isothiocyanate (FITC) Annexin V apoptosis detection kit I (BD Biosciences, Franklin Lakes, NJ, USA) according to the manufacturer’s recommendations, as described previously [29].

### 4.6. Cell Cycle Analysis Using Propidium Iodide (PI) Staining

After 24 h in serum-free media until cells reached 70–80% confluency on 6-well plates, the cells were treated with gentisyl alcohol (0 μM, 2 μM, 5 μM, 10 μM, and 20 μM) in a dose-dependent manner for 48 h at 37 °C in a CO_2_ incubator. Supernatants were transferred from culture dishes to collection tubes and analyzed using flow cytometry after staining with PI, as described previously [29].

### 4.7. TUNEL Assay

The ES2 and OV90 cells (3 × 10^4^ cells) were seeded on confocal dishes. After cells reached 70–80% confluency, they were treated with gentisyl alcohol at 20 µM and incubated for 24 h at 37 °C in a CO_2_ incubator. Subsequently, the cells were air-dried and fixed with 4% paraformaldehyde in PBS and incubated further for 1 h at room temperature. After rinsing and permeabilizing the cells, they were subjected to a TUNEL staining mixture using the In Situ Cell Death Detection kit (Roche) for 1 h at 37 °C in the dark, as described previously [29].

### 4.8. Detection of Mitochondrial Membrane Potential via JC-1

After treatment of gentisyl alcohol in a dose-dependent manner (0 μM, 2 μM, 5 μM, 10 μM, and 20 μM) for 48 h at 37 °C in a CO_2_ incubator, supernatants and adherent cells detached with trypsin- ethylenediaminetetraacetic acid (EDTA) were collected by centrifugation. The collected cells were resuspended in a staining solution that included 200× JC-1 and 1× staining buffer and were incubated at 37 °C in a CO_2_ incubator for 20 min. The stained cells were collected by centrifugation and washed once with 1× JC-1 staining buffer. After washing, the cells were centrifuged one more time and resuspended in 500 μL staining buffer. Fluorescence intensity was analyzed using a FACSCalibur (BD Bioscience).

### 4.9. Calcium Ion Concentration Assay in Mitochondrial Matrix

After treatment of gentisyl alcohol in a dose-dependent manner (0 μM, 2 μM, 5 μM, 10 μM, and 20 μM) for 48 h at 37 °C in a CO_2_ incubator, supernatants and adherent cells were collected by centrifugation. The collected cells were resuspended using 3 μM Rhod-2 AM (Invitrogen) diluted in Hank’s Balanced Salt Solution (HBSS) at 4 °C for 30 min. The stained cells were washed with HBSS and incubated at 37 °C for 10 min. Fluorescent intensity was analyzed using a flow cytometer (BD Bioscience).

### 4.10. Calcium Ion Concentration Assay in Cytosol

After treatment of gentisyl alcohol in a dose-dependent manner (0 μM, 2 μM, 5 μM, 10 μM, and 20 μM) for 48 h at 37 °C in a CO_2_ incubator, supernatants and adherent cells were collected by centrifugation. The collected cells were resuspended using 3 μM fluo-4 AM (Invitrogen) and incubated at 37 °C in a CO_2_ incubator for 20 min. After washing the stained cells with PBS, fluorescent intensity was analyzed using a flow cytometer (BD Bioscience).

### 4.11. Western Blot Analysis

The concentrations of proteins in whole-cell extracts after incubation with gentisyl alcohol (0 μM, 5 μM, 10 μM, and 20 μM) or a combination of gentisyl alcohol (20 μM) with each inhibitor (20 μM) were determined using the Bradford protein assay (Bio-Rad, Hercules, CA, USA), with bovine serum albumin (BSA) as the standard. Proteins were denatured, separated using 10% sodium dodecyl sulfate polyacrylamide gel electrophoresis (SDS-PAGE) gels, and transferred to nitrocellulose membranes, as described previously [29].

### 4.12. Statistical Analysis

Data for the proliferation assay were subjected to analysis of variance (ANOVA) according to the general linear model (PROC-GLM) of the statistical analysis system (SAS) program (SAS Institute, Cary, NC, USA) to determine whether there were significant differential effects on proliferation of ES2 and OV90 cells in response to treatments. Differences with a probability value of * *p* < 0.05 were considered to be statistically significant. Data are presented as the mean ± standard error of the mean (SEM) unless otherwise stated.

## 5. Conclusions

In this study, we first verified the anti-cancer effect of gentisyl alcohol on human ovarian cancer cells by the demonstration of cellular mechanisms. There are limited data regarding the effects of gentisyl alcohol in cancers and other diseases. We are the first researchers to suggest an application of gentisyl alcohol to ovarian cancer, thus revealing its possible use as an anti-cancer supplement. In addition, we demonstrated the molecular mechanisms regulated by gentisyl alcohol in ES2 and OV90 cells. However, further studies on the specific component/s of gentisyl alcohol that could have major anti-cancer effects remains to be elucidated. Such studies will ultimately assist in developing supplements and therapeutic agents against the progression of ovarian cancer in the future.
